# Supplementary material for: Observation of stable Néel skyrmions in cobalt/palladium multilayers with Lorentz transmission electron microscopy
Source: Nat Commun. 2017 Mar 10;8:14761. doi: 10.1038/ncomms14761 (PMC5353624; doi:10.1038/ncomms14761)
Supplement: Supplementary Information — Supplementary Figures, Supplementary Notes, and Supplementary References [file ncomms14761-s1.pdf]

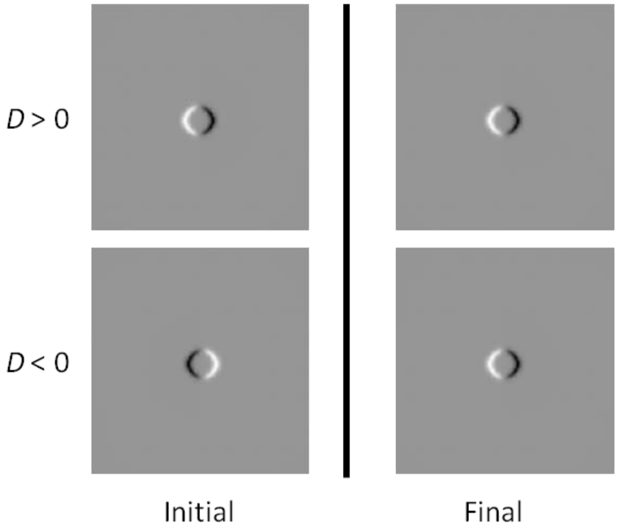

Supplementary Figure 1. **Chiral stability of Néel skyrmions**. Left: Initialized magnetic states with opposite chirality in a film with a Dzyaloshinskii-Moriya interaction strength, *D*, of 2.0 mJ m^{-2}. Right: final state following relaxation. The top skyrmion (a $D > 0$ skyrmion) does not change, while the lower skyrmion ($D < 0$) transforms into an identical configuration to that of the $D > 0$ skyrmion. White represents a positive horizontal *x*-magnetization, while black represents negative magnetization.

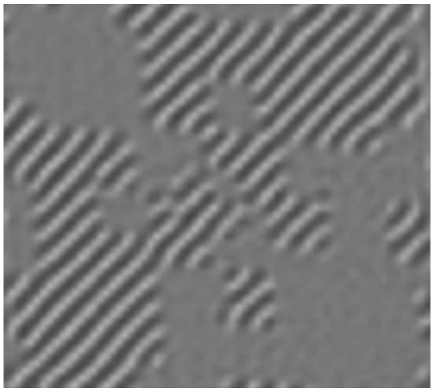

Supplementary Figure 2. **Simulated Lorentz image**. Simulated Lorentz image corresponding to the domain structure observed in Fig. 5d, with a tilt axis −45 degrees from the horizontal. The domain structure reproduces the dense chiral stripe phase observed in Fig. 7, with voids where Néel walls lie along the tilt axis.

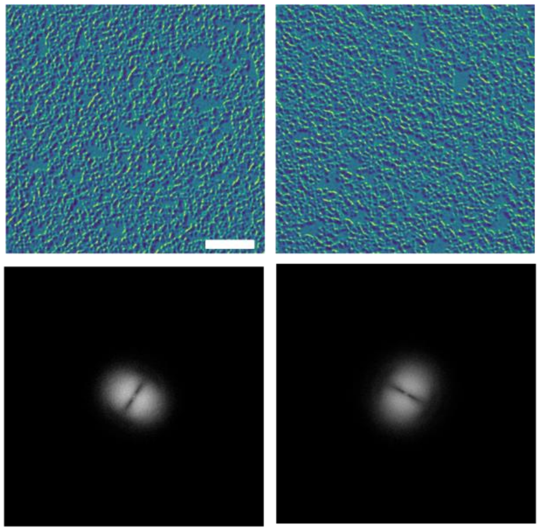

Supplementary Figure 3. **Tilt dependence of contrast in L-TEM**. Images (top) of the magnetic domain structure taken for two orthogonal tilt directions. The tilt direction may be determined by the directionality of the bright lobes in the FFT (bottom). However, the apparent directionality of the domain structure, evidenced by the FFT and observed clearly in the images, is solely due to the fact that contrast is only observed orthogonal to the tilt axis.

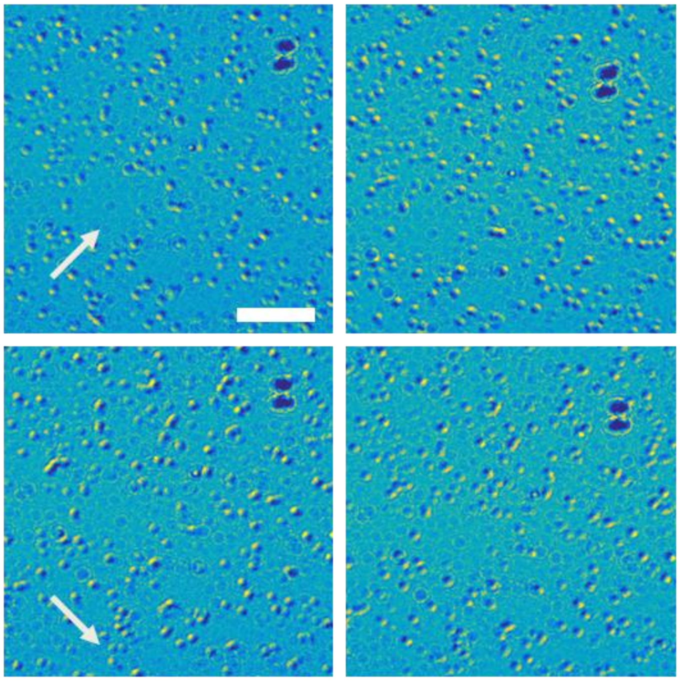

Supplementary Figure 4. **Different tilt directions in L-TEM**. Images taken at 4 different tilt directions following the nucleation of a dense skyrmion state. The angle for each is –30 degrees for the images on the left, and 30 degrees for the images on the right. The tilt axis for the top images is demonstrated by the arrow in the upper left image, while for the lower pair, the lower left image. The scale bar is 2 μm.

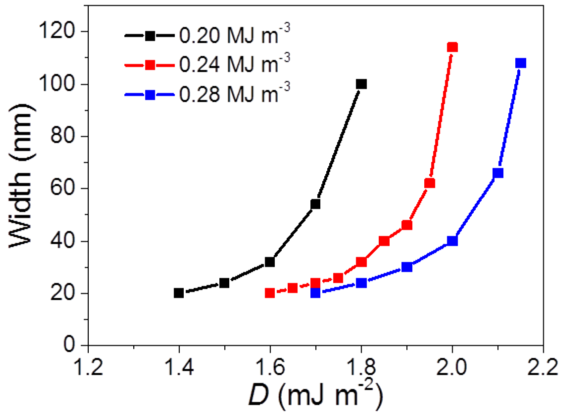

Supplementary Figure 5. **Anisotropy dependence of skyrmion stability**. Skyrmion size vs. D for with fixed A and varied effective anisotropy. Smaller anisotropy results in a lower D necessary to stabilize skyrmions, while the stable size range does not change significantly.

Supplementary Note 1. Chiral stability of Néel skyrmions in a system of fixed *D*

To verify that the Dzyaloshinskii-Moriya interaction (DMI) truly enforces a given chirality to a skyrmion in the films studied in this work, we have performed micromagnetic simulations on a system with D = 2.0 mJ m^{-2}, and the saturation magnetization (M_s), effective anisotropy ($K_{u,eff}$), and exchange stiffness (A) fixed at 880 kA m^{-1}, 0.24 MJ m^{-3}, and 10 pJ m^{-1} respectively. Two initial seed states were used, that with a skyrmion formed with D = 2.0 mJ m^{-2} and that with D = –2.0 mJ m^{-2}, which exhibited different chiralities, and then allowed to relax into the systems ground state. A stopping condition of dm/dt = 0.1 and a damping α = 0.5 were used. After relaxation, the D = 2.0 mJ m^{-2} state experienced no change to its magnetic structure. However, the D = –2.0 mJ m^{-2} state was found to be unstable, and rapidly transformed into the same spin texture observed in the D = 2.0 mJ m^{-2} skyrmion, as shown in Supplementary Figure 1. This demonstrates that, in the Co/Pd systems with strong DMI, only a fixed skyrmion chirality is stable, while the opposite chirality cannot exist even as a metastable state.

Supplementary Note 2. Estimating *D* by Kerr microscopy

In order to estimate the sign of the D in Co/Pd multilayers, as well as determine whether the strong DMI could be attributed to the Co/Pd multilayer and not just the Pt underlayer, we have utilized the method developed by Hrabec, et al.,[1] whereby a magnetic field is applied at a small angle to the film plane, and the velocity of domain wall expansion in the creep regime is measured via polar Kerr microscopy. The in-plane field, in concert with the DMI in the films, breaks the rotational symmetry of the expansion of a nucleated domain, with the in-plane field opposing expansion along one direction, and assisting it along the other. The direction of this asymmetry is determined by the sign of the DMI while its magnitude is determined by its strength. It should be noted that the multilayers which were used for skyrmion imaging possess too weak of perpendicular magnetic anisotropy for this measurement, as reversal is mediated not by domain wall creep expansion, but instead creation and propagation of nanoscale labrythine domain structures. Therefore, we utilized a structure with thinner Co and Pd layers, leading to significantly enhanced PMA.

We fit the velocity, $v(H) = v_0 \exp(-\zeta H_z^{-1/4})$, where v_0 is the characteristic velocity fitting parameter and H_z is a magnetic field applied out of the sample plane. In the presence of an in-

plane field, H_x, on a sample with non-zero DMI, $\zeta = \zeta_0 \left[\frac{\sigma(H_x)}{\sigma_0} \right]^{1/4}$, with ζ_0 being a scaling fit parameter and σ is the domain wall energy density as a function of H_x, given by

$$\sigma(H_x) = \begin{matrix} \sigma_0 - \frac{\pi^2 \Delta M_s^2 \mu_0^2}{8K_D}(H_x + H_{DMI})^2 & \text{for} & \mu_0 |H_x + H_{DMI}| < \frac{4K_D}{\pi M_S} \\ \sigma_0 + 2K_D\Delta - \pi\Delta M_s \mu_0 |H_x + H_{DMI}| & & \textit{otherwise} \end{matrix} . \quad (1)$$

$\sigma_0 = 2\pi\sqrt{AK_{0,eff}}$ is the Bloch wall energy density, $\Delta = \sqrt{A/K_{0,eff}}$ is the domain wall width, $K_{0,eff} = \mu_0 H_K M_s / 2$ is the effective magnetic anisotropy constant, and $4K_D / \pi M_s$ is the magnetic field required to stabilize a Neel type domain wall. $4K_D = \mu_0 N_x M_s^2 / 2$ is the domain wall anisotropy energy density. M_s is the saturation magnetization and H_K is the anisotropy field. A demagnetization coefficient of $N_x = 0.22$ and an exchange stiffness constant of A = 10 pJ m^{-1} are used in the fitting. Following this method, H_{DMI} is extracted and converted to a value of D by the relation $D = \mu_0 H_{DMI} M_S \Delta$. The fitting results, as well as representative images, are shown in Fig. 6.

Supplementary Note 3. Tilt dependence of contrast in L-TEM

As was mentioned in the main text, the fact that the contrast depends strongly on the tilt direction can give rise to artifacts in the imaging that can result in misinterpretations of the observed spin structure. To emphasize this, we utilized the domain pattern shown in Fig. 5d and simulated the contrast observed by tilting the sample 30 degrees from about an axis –45 degrees from the horizontal. Doing this produces contrast similar to that observed in the experimental reversal images shown in Fig. 7, with distinct voids appearing in which the chiral Neel domains lie parallel to the tilt axis. Results of the simulations are shown in Supplementary Figure 2.

This effect is especially apparent with intermediate magnetization states during experimental reversal measurements and present themselves as a perceived directionality to the magnetization reversal. In Supplementary Figure 3, we show the contrast obtained after application of a 50 mT field to a film pre-saturated in the +z direction. Imaging was performed at zero-field following field application. Both images appear to have a domain propagation direction orthogonal to the tilt axis, and this is also confirmed in the directionality of the FFT of

the image. However, summing the FFT's together results in no perceived directionality, as expected.

For a Néel skyrmion, however, due to its symmetry, the change in tilt from *x* to *y* only results in a rotation of the contrast. This is demonstrated in Supplementary Figure 4, wherein four images are taken along four different tilt directions.

Supplementary Note 4. Micromagnetic simulations of skyrmions and estimation of *D*

To estimate the magnitude of DMI in the Co/Pd films, we have performed micromagnetic simulations and determined at which values of *D* allowed for stable, zero field skyrmions. We utilized an M_s = 880 kA m^{-1} and an $K_{u,eff}$ = 0.24 MJ m^{-3}. A cell size of 2 nm × 2 nm × 6.7 nm was used, and simulations were started with an initialized skyrmion of 100 nm in extent. The system was then allowed to relax at zero field, and the width of the skyrmion was measured. As the skyrmion size varies significantly with *D*, this method allows for an approximation of its magnitude. It is well known that the skyrmion size and stability will also vary for a given exchange stiffness, *A*. In order to take this into account, we varied *A* from 7.5 – 12.5 pJ m^{-1}, near 10 pJ m^{-1} reported elsewhere for similar multilayer systems (i.e. Ref. [2]). At each *A*, we find a different range of values for *D* that result in zero field skyrmions (Fig. 5). For *A* = 7.5 pJ m^{-1}, this results in a stable range of *D* from 1.4 – 1.75 mJ m^{-2}, above and below which the skyrmion was no longer stable. However, below 1.7 mJ m^{-2}, the skyrmion size is significantly smaller than that found in our work. Increasing *A* increases the minimum and maximum values of *D* that result in stable skyrmions, while also increasing the maximum measured size before the skyrmion becomes unstable. Beyond the maximal value of *D* shown in Fig. 5, for each given *A*, the skyrmion state is no longer stable, and deforms into a chiral stripe domain. From these simulations, we conclude that our result matches with $|D|$ = 2.0 ± 0.3 mJ m^{-2}.

Supplementary Note 5. Anisotropy dependence of skyrmion stability

To understand the role that anisotropy, and that of its uncertainty in our determination of *D*, we performed micromagnetic calculations on a system identical to those which were used to identify *D* in the main text, except with a fixed *A* = 10 pJ m^{-1} and instead variable $K_{u,eff}$. We find that changes in $K_{u,eff}$ result in a shift of the range in which skyrmions are stable, with larger $K_{u,eff}$ requiring a larger *D*. However, we find that for small changes, this effect is quite small, as measurement error in typical magnetometry measurements of anisotropy is below 10%.

Simulations shown in Supplementary Figure 5 were performed over a range from 0.20 to 0.28 MJ m^{-3}. The stability band shows a clear shift relative to Fig. 5, however, the range of stable skyrmion sizes does not change significantly.

Supplementary References

1. Hrabec, A. *et al.* Measuring and tailoring the Dzyaloshinskii-Moriya interaction in perpendicularly magnetized thin films. *Phys. Rev. B* **90,** 020402 (2014).

2. Shaw, J. M. *et al.* Reversal mechanisms in perpendicularly magnetized nanostructures. *Phys. Rev. B* **78,** 024414 (2008).
